# Standing on the shoulders of microbes: How cancer biologists are expanding their view of hard‐to‐kill persister cells

**DOI:** 10.15252/msb.202211168

**Published:** 2022-07-22

**Authors:** Yaara Oren

**Affiliations:** ^1^ Department of Human Molecular Genetics & Biochemistry, Sackler Faculty of Medicine Tel Aviv University Tel Aviv Israel

**Keywords:** Cancer

## Abstract

Similar to persister bacterial cells that survive antibiotic treatments, some cancer cells can evade drug treatments. This Commentary discusses the different classes of persister cells and their implications for developing more efficient cancer treatments.

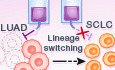

Similar to persister bacterial cells that survive antibiotic treatments, small populations of cancer cells can evade drug treatments and cause recurrent disease. This Commentary discusses the different classes of persister cells and their implications for developing more efficient cancer treatments.

In 1944, Joseph Bigger, a lieutenant‐colonel in the British Royal Army Medical Corps, reported a peculiar population of bacteria that could survive very high concentrations of penicillin (Bigger, 1944). He termed these hard‐to‐kill cells “persisters” and argued they might explain the limited success of penicillin in curing infections. At the time, 16 years after antibiotics revolutionized bacterial infection treatment, this was a groundbreaking hypothesis as it was largely believed that partial killing was mostly due to inadequate blood supply or tissue barriers. Later on, the understanding that cell‐intrinsic properties may contribute to transient drug tolerance sparked research aimed at targeting microbial persister cells. In a seminal paper, Sherma and colleagues (Sharma *et al*, 2010) showed that reversible cell‐intrinsic resistance can also be observed in cancer cells in response to therapy. Similar to bacterial persisters, these cancer persister cells gave rise to a drug‐sensitive cell progeny following a short “drug‐holiday” and did not harbor any known resistance‐mediating alteration mutation. However, in contrast to microbial persisters that are largely dormant, a small fraction of cancer persister cells were able to resume proliferation even under continued drug treatment. Understanding the similarities and differences between cancer and microbial persister cells is pivotal to devise approaches to eliminate them (Fig 1).

**Figure 1:**
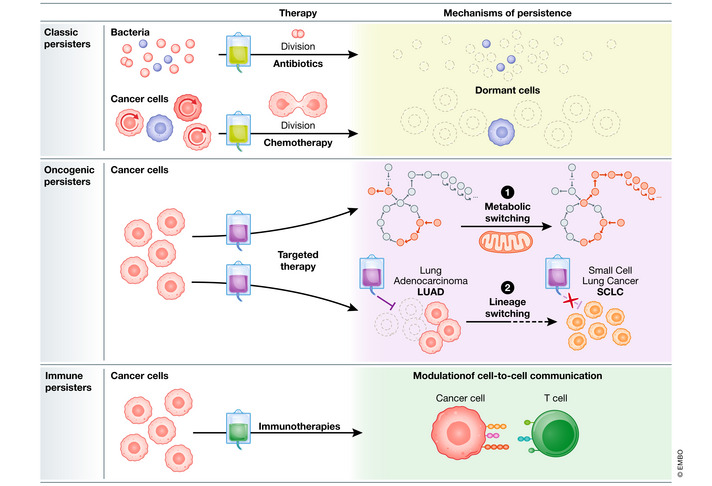
Different persister classes (A) Classic persisters, (B) targeted‐persisters, and (C) immune‐persisters. The mechanism of escape is dependent on the mode of action of the drug. While classical persisters are common to both bacteria and cancer cells, other persister classes are cancer‐specific and are associated with the ability of cancer cells to probe a wide range of cells states and lineage trajectories.

So why can some bacteria persist in the face of therapy? The answer largely lays in the mode of action of antimicrobial drugs. Penicillin and newer generation antibiotics target bacterial cell division. As such, if the bacteria are dormant or reside in a low metabolic state, they are unafflicted by the drug. Dormant bacteria are frequently resistant to multiple stressors and drugs making them difficult to eradicate even with a very aggressive treatment. Unsurprisingly, similar phenomena are observed in the context of chemotherapy treatments in cancer. Like antibiotics, early cancer therapies were largely based on drugs that target highly proliferative cells. Sustained proliferation in the absence of external stimuli is one of the hallmarks of cancer. Because cancer cells divide more frequently than most normal cells, they are more likely to be killed by chemotherapy treatment. As both antibiotics and chemotherapy treatments target proliferating cells, it is not surprising that cell dormancy was linked to cell persistence in both cases. “Classical” nondividing persister cells have been implicated in treatment failure both in cancer and in microbial infections and are thought to provide a reservoir for subsequent relapse events.

In the last 20 years, a new class of cancer drugs, called targeted therapies, have emerged and revolutionized patient care. Unlike chemotherapies or antibiotics, these drugs do not target proliferating cell *per se* but rather act on specific molecular targets associated with cancer. For example, some targeted therapies target proteins that are more abundant on the surface of cancer cells compared with that of normal cells. While slow proliferation has also been implicated in tolerance in the context of targeted therapy, multiple additional mechanisms are at play, which are not characteristic of microbial persister cells. For instance, oncogene‐targeted therapies are taking advantage of the acquired dependence of a cancer cell on the activity of a single oncogenic gene product. As many oncogenes control cell metabolism (Levine & Puzio‐Kuter, 2010), for example by regulating glucose uptake, drugs that target oncogene addiction can have profound effects on metabolism. In line with this, oncogenic‐persisters, for example, persisters that escape killing by oncogene‐targeted therapies show higher levels of fatty acid oxidation (Oren *et al*, 2021). This shift away from the “Warburg” glycolytic state into a more mitochondrially active energy production state, which resembles non‐transformed cells, might indicate the release from oncogenic addiction. Importantly, this shift does not lead to overall lower metabolic activity and in some cases might even allow persisters that were arrested to resume cell cycle in the presence of a drug. This high modularity is possible in cancer cells as they can, under certain conditions, tap into a vast space of cellular states that reflect different tissues and developmental trajectories. Cancer persister cell plasticity is perhaps best exemplified by phenotypic transformation from non‐small‐cell lung adenocarcinoma to small‐cell lung cancer upon prolonged treatment with EGFR inhibitors (Shaurova *et al*, 2020). Such lineage switching accounts for up to 14% of acquired resistance to EGFR‐targeted therapy. Clinical data of relapsed patients strongly support the hypothesis that this transformation happens via persister cells that were able to withstand EGFR therapy. Taken together, these observations show that cancer persister cells can circumvent oncogenic withdrawal by adopting alternative cell states. Notably, these changes do not necessarily require any genetic alteration and in theory can be reversible and potentially mediated by microenvironment signaling.

The most recent addition to the cancer‐fighting arsenal are immunotherapies designed to boost immune responses. Immune‐persisters, cells that can evade immune response, have been reported in multiple cancer types and are thought to underlie the late relapse frequently observed in patients (Shen *et al*, 2020). While tumor dormancy might play a role in this context as well, it is interesting to note that immune evasion can be achieved by modulating immune checkpoint molecules without any need to suppress cell proliferation. Furthermore, in the case of CAR T‐cell therapy, a class of immunotherapy that is based on revamped T cells, persistence might be viewed as a dynamic cell‐to‐cell communication process. It was shown that to elicit killing a cancer cell has to have multiple interactions with a T cell (Weigelin *et al*, 2021). This multihit sequential process that can take more than an hour *in vivo* may allow cancer cells to modulate the cytotoxic T cell in a way that would favor their persistence. Hence, understanding what underlies T‐cell phenotypes might as be as important as studying the cancer persister cells they are targeting.

The holy grail of the persister filed is finding ways to target these drug‐tolerant cells in a manner that would prevent disease recurrence. However, given at least three classes of persisters have been already reported, and more are expected to arise as we continue to expand our therapeutic toolbox, would it even be possible to implement a single approach to eliminate them? Studies that searched for a magic bullet that could eliminate persister cells were largely based on the hope that persister cells would be less heterogenous than the drug‐naïve cell population they were derived from (Cabanos & Hata, 2021; Hangauer *et al*, 2017). If such convergence on similar cell states exists upon treatment, it simplifies the need to combine multiple drugs to eliminate the entire cell population. Unfortunately, it seems that persister cells can come in multiple forms and that distinct persister phenotypes may coexist in a single tumor. The major drivers of this heterogeneity currently remain unclear and may include tumor lineage, treatment type, or a combination of both. Moreover, it is unknown if the heterogeneity in persister phenotypes can be predicated based on the drug‐naïve population and how these diverse persister fates are associated with clinical outcomes. Understanding persister heterogeneity is critical as the simplistic approach of trying to eliminate as many persister cells as possible, assumes that all cells are equally pathogenic, which might not be the case if only a subset of them are able to contribute to relapse. Furthermore, persister cells might differ in their aptitude to give rise to cells that harbor a resistance‐mediating mutation. Such differences in evolvability must be considered when weighing possible treatments. Answering these questions would be key to devising effective therapeutic approaches to eliminate persister cells. In the last century, the study of microbial persistence had provided important insights into how to fight infections. Hopefully, in the years to come, we will build upon this valuable knowledge foundation and expend it to devise better ways to fight cancer.

## Disclosure and competing interests statement

The author declare that she has no conflict of interest.
